# New Molecular Targets for Antidepressant Drugs

**DOI:** 10.3390/ph14090894

**Published:** 2021-09-02

**Authors:** Johannes Kornhuber, Erich Gulbins

**Affiliations:** 1 Department of Psychiatry and Psychotherapy, University Hospital, Friedrich-Alexander-University of Erlangen-Nuremberg, 91054 Erlangen, Germany; 2 Department of Molecular Biology, University of Duisburg-Essen, 45117 Essen, Germany; erich.gulbins@uk-essen.de; 3 Department of Surgery, University of Cincinnati, Cincinnati, OH 45267, USA

**Keywords:** acid sphingomyelinase, ceramide, sphingomyelin, FIASMA, antidepressant drug, lysosome, neurogenesis, hippocampus, autophagy, brain-derived neurotrophic factor (BDNF)

## Abstract

Major depressive disorder (MDD) is a common and severe mental disorder that is usually recurrent and has a high risk of suicide. This disorder manifests not only with psychological symptoms but also multiple changes throughout the body, including increased risks of obesity, diabetes, and cardiovascular disease. Peripheral markers of oxidative stress and inflammation are elevated. MDD is therefore best described as a multisystem whole-body disease. Pharmacological treatment with antidepressants usually requires several weeks before the desired effects manifest. Previous theories of depression, such as the monoamine or neurogenesis hypotheses, do not explain these characteristics well. In recent years, new mechanisms of action have been discovered for long-standing antidepressants that also shed new light on depression, including the sphingolipid system and the receptor for brain-derived neurotrophic factor (BDNF).

## 1. Introduction

For many clinically successful drugs, new and relevant mechanisms of action have been discovered over time. For example, we have demonstrated relevant antagonistic effects of memantine, amantadine and budipine, which until then had been considered dopaminomimetic substances, on N-methyl-D-aspartate (NMDA) receptors [1,2,3]. A new mechanism of action has only recently been demonstrated for the very well established and effective drug metformin in the treatment of type 2 diabetes [4]. This is also true for antidepressants, which have been used successfully to treat major depressive disorder (MDD) since 1957 [5]. These compounds were initially used clinically without the knowledge of their molecular targets. Over time, their mechanisms of action were initially discovered to be the inhibition of monoamine oxidases and the inhibition of monoaminergic transporters. Later, other molecular mechanisms were discovered, such as the inhibition of postsynaptic serotonin receptors, the inhibition of presynaptic alpha 2 receptors, and the activation of melatonergic receptors.

In recent years, a number of new antidepressants with novel mechanisms of action have been developed and clinically evaluated. The inhibition of NMDA receptors by esketamine shows a rapid antidepressant effect [6]. However, NMDA receptor inhibition as an antidepressant mode of action is in doubt, since a ketamine metabolite has low affinity for the NMDA receptor but has antidepressant activity [7]. A single i.v. administration of brexanolone, a neuroactive steroid (SAGE-547) with gamma-aminobutyric acid (GABA)_A_ receptor-positive allosteric modulatory activity, shows a rapid antidepressant effect that lasts more than a week in postpartum depression [8,9]. Zuranolone, an orally available neuroactive steroid with a comparable mechanism of action (SAGE-217), also shows antidepressant effects on MDD [10] and postpartum depression [11]. In addition, there are a number of other compounds, each with different molecular targets and potential antidepressant effects, that are currently being evaluated [12].

In parallel with the development of new compounds and the exploitation of new molecular targets, important cellular and systemic mechanisms of action of antidepressants have been discovered, such as neurogenesis [13], neuroplasticity and inflammation. However, it is not yet clear through which molecular targets neurogenesis, neuroplasticity and inflammation are affected.

The focus of this review is relatively narrow. We discuss only novel molecular targets other than the previously described targets for the groups of licensed antidepressants (i.e., Group N06A in the ATC system), and we only consider developments during the last 10 years. The mechanisms should apply not only to individual substances but also to many antidepressants, even structurally different ones. We discuss in detail the effect of antidepressants on the sphingolipid system and the tyrosine kinase receptor 2 (TRKB) receptor for BDNF.

## 2. MDD as a Whole-Body Multisystem Disease

**Figure 1:**
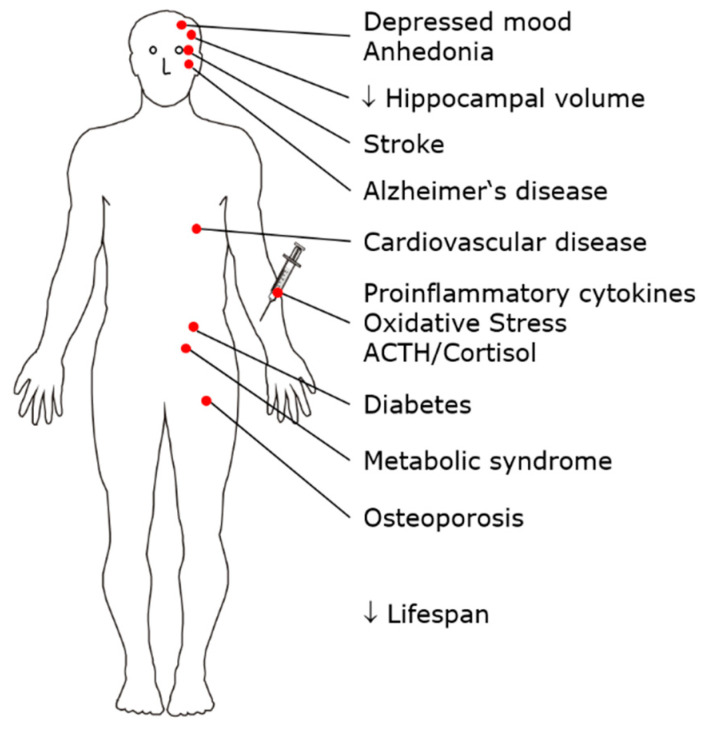
MDD is a risk factor for the development of multiple disorders and diseases, such as metabolic syndrome, osteoporosis, and cardiovascular disease. MDD can therefore be conceptualized as a multisystem, whole-body disease.

MDD is a common mental disorder with a lifetime prevalence of approximately 10%. Patients suffer a high overall burden of disease due to severe impairment during each episode of illness, long individual episodes of illness, an often early age of onset, and an often recurrent course [14]. MDD is a risk factor for the development of cardiovascular disease, diabetes, metabolic syndrome, osteoporosis, Alzheimer’s disease, and hippocampal atrophy. The adrenocortical stress axis is activated, and changes in serum lipids occur; peripheral proinflammatory cytokines, including interleukin-1β (IL-1β), markers of oxidative stress and inflammation, and serum phospholipase A2 activity are increased. Independent of suicide, life expectancy is reduced in patients with MDD. Therefore, MDD is considered not only a mental disorder but also a multisystem whole-body disease [15,16,17,18,19] (Figure 1).

The diagnostic criteria for major depression according to the DSM-5 [20] include predominantly psychological symptoms apart from fatigue and weight change. The physical illnesses listed here, which occur frequently as a follow-up to depression, do not belong to the diagnostic criteria.

## 3. Current Pharmacological Treatments for MDD

The group of antidepressants defined in the ATC system (N06A) includes substances with very different structures and mechanisms of action. According to the current view, antidepressants can act, for example, by modulating central monoaminergic transmission and NMDA receptors or via the melatonergic system. Antidepressants are more effective than placebos in treating major depression, and amitriptyline has the highest efficacy [21]. Antidepressants are particularly beneficial for severe depression [22]; however, see [23]. The effect size of electroconvulsive therapy (ECT) in the treatment of MDD is significantly higher than that of antidepressant drugs [24]. Antidepressants typically have to be taken for several weeks before an antidepressant effect occurs. This delayed onset of action is called therapeutic latency. Early improvement is often seen in medication responders, and the absence of such early improvement predicts poor clinical response. It takes 6–12 weeks for the maximal therapeutic effect to be achieved [25,26,27].

There are a number of unmet needs in the pharmacological treatment of MDD. The potential for optimization exists with regard to low remission rates and high treatment resistance, therapeutic latency, side effects and drug-drug interactions. Currently, there are limited approaches to individualizing pharmacologic therapy. The molecular mechanisms leading to depression are not sufficiently understood. As a result, important characteristics, such as the multisystem and whole-body nature of MDD, remain unexplained and are therefore not treatable in a targeted manner.

## 4. Neurobiological Hypotheses of Depression

MDD is treated with pharmacologic and nonpharmacologic methods; these treatments include antidepressants, sleep deprivation, physical activity, and ECT. The monoamine hypothesis of depression [28], which was established over 50 years ago, is based on the finding that the majority of currently used antidepressants interact with monoamines, particularly the 5-hydroxytryptamine (5-HT) system. The monoamine hypothesis of depression inadequately explains the typical phenomena of depression. The effect of antidepressants is not clearly correlated with the affinity of these drugs for monoamine transporters; tianeptine is an example that an antidepressant effect can be achieved not only by inhibiting but also by activating serotonin transporters [29]. The rapid effect of antidepressants on monoamine transporters does not match their therapeutic latency. The monoamine hypothesis also poorly explains the typical diurnal fluctuations in mood and the higher prevalence of depression in women than in men. Therefore, alternative depression hypotheses have been developed, including the neuroplasticity hypothesis [30], the neuroendocrine hypothesis, and the inflammation/cytokine hypothesis [19].

Decreased hippocampal neurogenesis is thought to be a key element in the pathophysiology of depression [13,31,32,33]. Consistent with this finding, chronic stress reduces neurogenesis in rodents and leads to hippocampal atrophy in humans. This effect can be normalized by the administration of antidepressants for 2–3 weeks, matching their therapeutic latency [13,31,32,33]. In stress-induced models of MDD, antidepressants have been shown to increase cell proliferation and neurogenesis in primary neuronal cultures in vitro and in the hippocampus of adult rodents in vivo and to improve behavior [13,31,32,33]. In contrast, the inhibition or ablation of neurogenesis, such as by selective irradiation of the hippocampus, does not lead to MDD [13]. Similarly, high-dose irradiation of the skull and brain in leukemia patients does not necessarily trigger MDD [34]. Moreover, ECT, sleep deprivation and some recently developed drugs, such as ketamine, have rapid therapeutic effects [35,36], which are inconsistent with the prolonged duration of neurogenesis and maturation as prerequisites for antidepressant therapy. Thus, it is still unclear how antidepressants work. We have shown that a lipid-based pathway is also involved in the pathology of depression and have identified the ceramide system as a novel antidepressant target [37,38].

## 5. The Sphingolipid System

Sphingomyelin is cleaved by acid sphingomyelinase (ASM) to form phosphorylcholine and ceramide. ASM is found in the lysosomes of all cells; since lysosomes constantly fuse with the plasma membrane, ASM is also found on the cell surface [39,40]. Sphingolipids are not only structural components of membranes; some sphingolipids play important roles as signaling molecules in cellular processes, also in the central nervous system (CNS) [41]. Ceramide, ceramide 1-phosphate (C1P), sphingosine, and sphingosine 1-phosphate (S1P) are bioactive sphingolipids that regulate processes such as cell growth, cell death, adhesion, migration, inflammation, and angiogenesis. The sphingolipid-rheostat model ascribes opposite roles for these compounds in cellular survival signaling: ceramide is a cell death activator, whereas S1P promotes survival [41]. A phosphorylation reaction converts ceramide and sphingosine into their antagonistic counterparts. However, the pro- versus anti-survival roles are not always rigid [41].

### 5.1. Altered Biophysical Properties of the Plasma Membrane

Increased ASM activity increases the ceramide level in the cell membrane. Ceramide is very hydrophobic and aggregates spontaneously in the cell membrane into large, hydrophobic rafts [39], thereby altering membrane biophysics, membrane fluidity, membrane fusion and budding, and the distribution of receptors on the membrane surface [39]. These ceramide-rich rafts play important roles in the cellular stress response [40,42,43,44]. This finding will be explained here using transient receptor potential cation channel subfamily C member 6 (TRPC6) as an example. These receptors are expressed in the plasma membrane and allow the passage of calcium and potassium currents [45]. TRPC6 is involved in the signaling of brain-derived neurotrophic factor (BDNF) [46], which is a pathophysiologically relevant neuropeptide in depression and stress disorders [47]. Hyperforin, a component of St. John’s wort used to treat mild to moderate depression [48] activates TRPC6 channels. The inhibition of ASM reduces ceramide abundance in the cell membrane, preventing clustering of TRPC6 in lipid rafts and thus altering TRPC6 function [49].

### 5.2. Direct Effects of Sphingolipids on Membrane Proteins

In addition to influencing the biophysical properties of the cell membrane and forming ceramide-rich rafts, sphingolipids can also interact directly with the transmembrane structures of proteins [50,51]. Both alterations in the biophysical properties of the cell membrane and the direct interactions of sphingolipids with membrane proteins can change the function of receptors and ion channels within the cell membrane.

### 5.3. Mechanisms That Activate the ASM/Ceramide System

Intracellular ceramide levels can be increased by various stimuli that activate the de novo synthesis pathway, the ASM pathway, or both. One of the major activators of ceramide formation is oxidative stress induced by reactive oxygen species (ROS) and reactive nitrogen species (RNS) [52,53]. In the context of depression, it is relevant that ROS and RNS, among others, are also generated by endogenous sources, such as proinflammatory cytokines (e.g., IL-1, IL-6, and TNF-α). Conversely, ceramides induce oxidative stress and NO production in endothelial cells [54].

The ASM/ceramide system has been intimately linked to the generation of ROS. In particular, in macrophages and endothelial cells, it was shown that ceramide-enriched membrane domains cluster components of NADPH oxidase; this clustering is a prerequisite for the correct assembly and function of NADPH oxidase and, thus, the release of ROS [55,56]. Ceramide has also been shown to accumulate in mitochondria, such as during aging [57]. It is possible that mitochondrial ceramide also regulates mitochondrial ROS generation during stress and MDD, although this hypothesis needs to be investigated.

### 5.4. Functional Inhibitors of ASM (FIASMAs)

Since the 1970s, weak organic bases such as desipramine have been shown to inhibit ASM activity [58,59,60,61,62]. ASM is bound to intralysosomal membranes and is thereby protected from proteolytic inactivation. Weak bases diffuse into lysosomes and are trapped after being protonated, which can lead to a 1000-fold intralysosomal accumulation of weak bases [63]. Weak bases also localize to other acidic subcompartments of the cell membrane and thus inhibit ASM not only in lysosomes but also in specific domains of the cell membrane.

The functional inhibition of ASM requires few structural conditions; molecules must contain a lipophilic part that integrates into the inner lysosomal membrane, a short spacer, and a charged tertiary amine group that displaces ASM from the inner lysosomal membrane, leading to proteolytic degradation of the enzyme in the lysosomal lumen [64,65]. Thus, weak bases do not directly inhibit ASM but enable the functional inhibition of ASM. We have proposed the acronym FIASMA (functional inhibitor of acid sphingomyelinase) for a compound in this large group of drugs [38]. The functional inhibition of ASM is not achieved through a specific molecular target site, such as a drug-receptor interaction, but rather via general physicochemical properties of the drugs that lead to their accumulation in acidic regions and incorporation into the membrane, with a subsequent change in membrane charge. All FIASMAs identified to date have at least one basic nitrogen atom and a medium to high logP value, and most have a molecular weight less than 500. Due to their ability to cross the blood–brain barrier, all FIASMAs reach the brain. Conversely, not all lipophilic weak bases are FIASMAs; for example, chloroquine is not a FIASMA [62]. We have identified a number of new FIASMAs (e.g., fluoxetine, fluvoxamine, maprotiline, nortriptyline, orphenadrine, sertraline, dextromethorphan, emetine, and triflupromazine) [61,62]. Many of these compounds are U.S. Food and Drug Administration (FDA)-approved for human use, are inexpensive and safe and are therefore potentially rapidly available for drug repurposing.

The accumulation in lipophilic membranes and acidic intracellular compartments explains the high volume of distribution of FIASMAs. The FIASMA amitriptyline will be described in more detail as an example. Due to its high lipophilicity and weak basicity, amitriptyline highly accumulates in tissue compartments, such as lysosomes [63], resulting in a high volume of distribution (Vd = 16 L/kg, www.drugbank.ca; accessed on 1 August 2021) and high tissue concentrations in all organs [66,67,68].

FIASMAs are cationic amphiphilic drugs (CADs) that act within acidic intracellular compartments to affect not only ASM but also the catalytic activities of other lysosomal enzymes. This can lead to the side effect of phospholipidosis (PLD), which is a deleterious increase in phospholipids in lysosomes. FIASMAs differ only slightly in their physicochemical properties, but their effects on ASM activity and the induction of lysosomal phospholipid content vary significantly. Systematic chemical modifications to the model FIASMAs imipramine, desipramine, and fluoxetine may lead to other more suitable compounds with more pronounced inhibition of ASM and less induction of PLD. Small chemical changes to FIASMAs may therefore result in compounds with better antidepressant properties and fewer side effects than the parent compounds [69].

By inhibiting ASM, FIASMAs result in lower cellular concentrations of ceramides. Human studies have shown reduced ceramide concentrations in lung endothelial and nasal epithelial cells after treatment with 25–75 mg/day amitriptyline for 2–4 weeks [70,71].

## 6. Sphingolipids in MDD

### 6.1. A Priori Arguments for Involvement of the ASM/Ceramide System in MDD

There are several a priori arguments for the involvement of the ASM/ceramide system in the pathophysiology of MDD. (1) It has long been known that tricyclic antidepressant drugs such as desipramine functionally inhibit ASM. (2) This system gathers and amplifies a wide range of stress signals and thereby regulates important cellular functions [72]. (3) The ASM/ceramide system is evolutionarily conserved and is present in all eukaryotic cells; this system is therefore present in all cells in the body and not only in the brain. The ASM/ceramide system may therefore explain not only CNS involvement in MDD but also the peripheral comorbidities and consequences of MDD.

The systematic review by Dinoff et al. on the relationship between ceramides and depression summarizes the data through 2017. Preclinical and clinical studies have suggested the involvement of ceramides in the pathophysiology of depression, with a particularly strong association of the ceramides C18:0 and C20:0 with depression in humans [73]. Since then, a number of other preclinical and clinical studies have confirmed the link between ceramides and depression [74,75,76,77,78,79,80,81]. Furthermore, in a population-based cross-sectional study, environmental mastery, which is a resilience factor for depressive disorders, correlated negatively with total plasma ceramides [82]. However, because of the cross-sectional design of these clinical studies, causality cannot be inferred.

### 6.2. Behavioral Effects of Antidepressant Drugs Are Mediated by the ASM/Ceramide System

The hippocampus is one of the most interesting targets of antidepressant drugs. The hippocampus is important not only for learning and memory but also for emotional regulation. Patients suffering from depression and anxiety often show hippocampal atrophy [83], which is reversible through the intake of antidepressants. In this particular brain region, new neurons are constantly formed. Stress, which may induce a major depressive episode, strongly reduces neurogenesis. Importantly, antidepressant drugs increase adult neurogenesis. Inhibiting adult neurogenesis blocks the behavioral effects of antidepressant drugs. This effect was previously known [84]. We wondered whether the ASM/ceramide system is involved in these processes [37].

We have shown a central role of hippocampal ceramide in depression development. Lowering hippocampal ceramide by therapeutic concentrations of the antidepressants amitriptyline and fluoxetine normalizes hippocampal neurogenesis and behavior in mouse models of stress-induced depression. Genetic ASM deficiency abrogates these effects of antidepressants. In contrast, increasing ceramide levels such as by overexpressing ASM, blocking ceramide degradation, or directly injecting C16 ceramide into the hippocampus results in reduced neurogenesis and depression-like behavior, even in the absence of stress. The antidepressant-mediated reduction in ceramide concentrations via the inhibition of ASM normalizes these effects. Thus, lowering the ceramide concentration may be a key strategy in future antidepressant development [37]. The ability of subchronically applied FIASMAs to reduce hippocampal ceramide was confirmed with paroxetine [79].

Based on the crystal structure of ASM [85,86,87], direct inhibitors of ASM have been developed [80]. Substance 21b penetrates the cell membrane in vitro and crosses the blood–brain barrier in vivo after intraperitoneal administration. Substance 21b inhibits ASM in the cortex and hippocampus, induces hippocampal neurogenesis, and normalizes behavior in rat depression models. These data confirm ASM as a target for depression treatment and expand the potential compounds that can be used to include direct inhibitors of ASM [80].

### 6.3. Autophagy

#### 6.3.1. The Sphingomyelin-Ceramide System

Antidepressants such as amitriptyline or fluoxetine induce hippocampal autophagy [88]. Functional inhibition of ASM by these antidepressants leads to slow increases in sphingomyelin in lysosomes and Golgi membranes and an increase in ceramide in the endoplasmic reticulum (ER). Ceramide in the ER stimulates autophagy proteins, including Beclin, via a signal transduction cascade. These processes require at least 12 days of antidepressant treatment; in contrast, direct inhibition of sphingomyelin synthases (SMS) with tricyclodecan-9-yl-xanthogenate (D609) results in a rapid increase in ceramide in the ER, as well as the induction of autophagy and the amelioration of the biochemical and behavioral effects of stress-induced depression. Beclin inhibition abolishes the antidepressant effects of amitriptyline and D609 and induces cellular and behavioral effects similar to those seen in MDD [88]. Chronic unpredictable mild stress, chronic social defeat stress and corticosterone reduce autophagy markers in the rodent hippocampus [88,89,90,91,92]. Beclin activates autophagy [93], while spautin-1, an inhibitor of Beclin, reduces autophagy [94] and depression-like behavior and abrogates the effects of amitriptyline on depression-like behavior and neurogenesis [88].

These results argue for a central role of autophagy in the pathophysiology of MDD and the effect of antidepressants. Environmental stress and corticosterones induce autophagy; antidepressants such as amitriptyline or fluoxetine attenuate autophagy. A direct increase in ceramide levels in the ER induces autophagy much more rapidly than amitriptyline or fluoxetine. Drugs, such as D609 or derivatives thereof, that cause the rapid induction of autophagy could be a new class of rapidly acting antidepressants. D609 has also been shown to induce ceramide synthesis [95], which would further increase the ceramide concentration in the ER and promote the antidepressant effect of D609. In summary, these findings identify sphingolipid-driven autophagy as an important target for antidepressant treatments and provide a rationale for the development of novel antidepressants that act within days [88].

The substance D609 used in previous investigations inhibits both SMS1 and SMS2 [96,97], which have different subcellular localizations and tissue distributions. While specific inhibitors have been described for SMS2, corresponding specific compounds for SMS1 are lacking [98,99,100]. Currently, it is not known which of the two enzymes is a better target for affecting autophagy; thus, it is unclear whether SMS1 or SMS2 is the better target for antidepressant therapy or whether inhibiting both enzymes is necessary. These questions can be better answered if specific inhibitors are available for SMS1. In this context, it is interesting to note that the inhibition of SMS2 ameliorates metabolic alterations that are common in depression: Ly95 has antidiabetic and antiatherosclerotic effects [100,101]. This allows for the hypothesis that SMS2 inhibitors also have an antidepressant effect. SMS1 and SMS2 are encoded by the SGMS1 and SGMS2 genes, respectively. In the mouse brain, these genes are unevenly expressed regionally; for example, these genes are particularly strongly expressed in serotonergic and noradrenergic neurons (http://mousebrain.org/genesearch.html; accessed on 1 August 2021). This brain regional distribution suggests an antidepressant effect for both enzymes and perhaps even an optimal antidepressant effect through the simultaneous inhibition of both enzymes.

Ceramide generated in mitochondrial membranes has also been shown to bind microtubule-associated protein 1 light chain 3 β-lipidation, forming LC3B-II and thereby promoting mitophagy [102]. The dysregulation of mitophagy by increased concentrations of ceramide might disturb cellular energy metabolism and thereby contribute to MDD. However, because mitochondrial ceramide is reportedly generated by ceramide synthases, this pathway would not be directly regulated by FIASMAs, but indirect regulation similar to the studies described above might be possible.

#### 6.3.2. Autophagy and the Clinical Characteristics of MDD

The results regarding autophagy are consistent with important clinical observations. First, the typical symptomatology of MDD is compatible with a reduction in autophagy. Adult hippocampal neurogenesis is reduced under stress and in animal models of depression [13,31,32,33,88], which might explain the hippocampal atrophy observed in MDD patients [83]. This finding is compatible with the important role of autophagy in adult neurogenesis [88,103]. MDD is associated not only with psychological symptoms but also, as described above, with a number of physical pathologies, such as cardiovascular disease. These peripheral symptoms cannot be explained by altered neurogenesis in the hippocampus alone, but they can be explained by alterations in autophagy in nonneuronal cells. Patients with severe MDD often experience marked weight loss, which returns to normal as depression improves. This finding is consistent with the finding of a lean phenotype in mice with genetically downregulated autophagy [104]. Many patients with MDD experience typical diurnal fluctuations with early waking, low mood, and decreased drive in the morning. Diurnal variations can hardly be explained by altered neurogenesis. However, these diurnal fluctuations match the 24 h rhythm of autophagy, with a nadir at 1:00 am [105]. The life expectancy of patients with MDD is reduced, even independent of suicide. This finding is consistent with the expected effects of reduced autophagy. The upregulation of autophagy extends lifespan in *Caenorhabditis elegans* and *Drosophila*, and autophagy-stimulating drugs, such as rapamycin, extend lifespan in rodents [106,107].

Second, predisposing factors for MDD are also consistent with a reduction in autophagy. Genetic predisposition to MDD may be partially explained by an effect on autophagy [108]. Older individuals are more likely to suffer from depression [109]. This finding is consistent with the finding of decreased autophagy in aging cells [106,110,111], which is probably also true in the brain [112]. Women are more likely to suffer from depression than men, but the reasons for this sex difference are not fully understood. X-chromosomal mechanisms and sex steroid hormones affect autophagocytosis [113]. In human umbilical endothelial cells (HUVECs), female cells show reduced autophagy [114]. Therefore, it is possible that autophagocytotic mechanisms are involved in the higher prevalence of depression in women than in men. However, corresponding studies on human neuronal cells are lacking. Stress is a risk factor for the development of MDD. Stress induced by corticosterone, chronic social defeat stress or chronic unpredictable mild stress, reduces neuronal autophagy; antidepressant treatments improve autophagy in these models [88,89,90,91,92].

Third, the effects of antidepressant therapies are also consistent with an autophagy-enhancing effect. Stimuli that promote autophagy counteract MDD. Many publications report an increase in autophagy with the application of antidepressants [115,116]. Lithium is used therapeutically in patients with MDD to augment the effects of antidepressants. This practice is consistent with the autophagy-inducing effect of lithium [117]. The fast-acting antidepressant ketamine induces autophagy [118]. ECT rapidly and transiently induces autophagy in the hippocampus [119], consistent with the rapid and transient antidepressant effect in patients. The antidepressant effects of physical activity [120,121] are compatible with increased autophagy [122,123,124]. Mediterranean and other healthy diets have preventive and therapeutic effects on future [125,126] and current [127] depressive episodes. This effect may be at least partially mediated by an effect on autophagy. While studies on the effect of the Mediterranean diet with its complex composition on autophagy are lacking, there have been findings on the individual components of this diet: polyphenols found in wine or olive oil and omega-3 fatty acids found in fish can induce autophagy [128,129]. Autophagy dysfunction contributes to the progression of pathologies that can be prevented with a Mediterranean diet. The downregulation of autophagy induces atherosclerosis and cardiovascular disease [130]. Autophagy is necessary for efficient cardiomyocyte development and function (literature in [129]). In addition, autophagy also plays an essential role in controlling the inflammatory response of macrophages [131]. Short-term caloric restriction triggers autophagy [132] and may have antidepressant effects on humans [133] and antidepressant-like effects on mice [134].

Fourth, the therapeutic latency of antidepressants such as amitriptyline or fluoxetine is explained through the backward effects after inhibiting ASM by the slow accumulation of the precursor substance sphingomyelin in the lysosome and ceramides in the ER. Even in the case of genetic ASM deficiency, sphingomyelin accumulates only slowly in lysosomes [115,135]. Apparently, the increase in lysosomal sphingomyelin induced by a pharmacological or genetic decrease in ASM activity can be slowed for a limited time by compensatory mechanisms. As described above, the slow increase in sphingomyelin induces autophagy via intermediate steps [88].

In summary, the associations between important clinical characteristics of depression and autophagy compiled here support an important role of autophagy in the development and treatment of depression.

## 7. Sphingolipids as Therapeutic Targets

Sphingolipid metabolism offers multiple opportunities for pharmacological interventions. The limitation is that intervention at a single node in this highly regulated metabolic system can affect the entire metabolic network in potentially unexpected ways. Whenever we make even small changes within a complex system, we risk dramatic unintended consequences. In addition to the expected “forward effects”, “backward effects” must also be considered. For example, FIASMAs inhibit ASM in acidic intracellular domains and thus reduce the product ceramide as a forward effect. However, the inhibition of ASM also leads to the slow accumulation of the precursor sphingomyelin in lysosomes and Golgi membranes and of ceramide in the ER, which in turn activates an autophagy cascade [88]. These consequences occur even though FIASMA-mediated inhibition of ASM is incomplete. In the case of FIASMAs, both the forward effects and the backward effects are therapeutically useful in terms of an antidepressant effect. This is a stroke of luck that cannot be expected with every therapeutic intervention.

## 8. Other Mechanisms of Action

**Figure 2:**
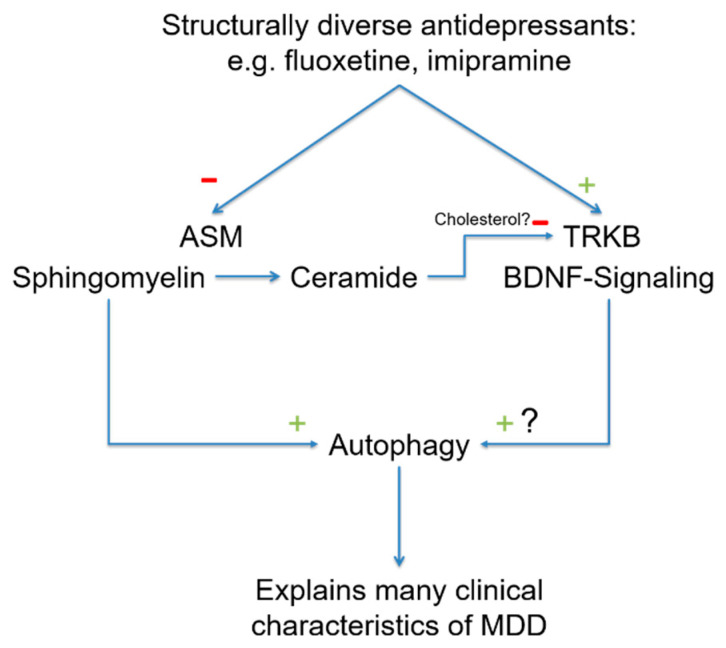
In recent years, two new molecular targets of antidepressants have been discovered. Many antidepressants functionally inhibit ASM, causing a reduction in ceramide in the cell membrane as a forward effect and a slow increase in sphingomyelin as a backward effect. Sphingomyelin increases autophagy via intermediate steps. Because ceramide in the cell membrane displaces cholesterol, antidepressants increase cholesterol by lowering ceramide levels. Cholesterol enhances TRKB dimerization and, thus, antidepressant binding. The BDNF-TRKB pathway induces autophagy, and so both signaling pathways converge here. A reduction in autophagy is observed in many of the phenomena associated with MDD, and increasing the rate of autophagy is likely to be useful in treating patients with MDD. Abbreviations: ASM, acid sphingomyelinase; BDNF, brain-derived neurotrophic factor; TRKB, tyrosine kinase receptor 2.

BDNF stimulates neuronal plasticity and induces antidepressant effects [136]. The receptor for BDNF is TRKB. Recently, it has been shown that antidepressants bind directly to TRKB and facilitate the binding of BDNF via allosteric modifications. This novel mechanism of action explains the effects of antidepressants on neuroplasticity [137]. This study demonstrated that antidepressants such as fluoxetine, ketamine and imipramine bind to a specific region in TRKB [137]. The binding of antidepressants to TRKB is facilitated by cholesterol. TRKB can sense cholesterol, which promotes TRKB dimerization. TRKB dimerization, in turn, promotes fluoxetine binding by forming a binding pocket for fluoxetine. The binding of antidepressants ultimately stabilizes the active conformation of TRKB and facilitates signaling via TRKB. The study also demonstrated that a point mutation in TRKB abrogates the binding of antidepressants [137]. Notably, in membrane domains, ceramide displaces cholesterol [138], and a reduction in membrane ceramides through ASM inhibition might be a prerequisite of FIASMAs binding to TRKB to stabilize TRKB signaling. Furthermore, it might even be possible that the binding of antidepressants to TRKB involves sphingomyelin and thereby prolongs FIASMA activity. In this model, antidepressants might act on ASM to increase sphingomyelin, which promotes autophagy and the direct or indirect binding of antidepressants to TRKB. TRKB has also been linked to the regulation of autophagy [139], and it is possible that both pathways converge at the level of autophagy (Figure 2).

## 9. Conclusions

Antidepressants have been used very successfully worldwide for over 60 years as safe drugs for treating depression. These drugs form a structurally diverse group of small molecular weight compounds, all of which are active in the CNS. A common feature of many antidepressants is their actions on monoaminergic transporters. However, important clinical characteristics of depression are not explained by the monoamine hypothesis, such as the latent onset of antidepressant effects, the typical diurnal variations with morning lows, the higher prevalence in women than in men, and the often observed increase in symptomatology in older individuals. Most importantly, the monoamine hypothesis does not explain the multisystem whole-body character of the disorder. In the last 10 years, new molecular targets have been discovered for long-established antidepressants. These targets are mostly associated with the effects of antidepressants on the sphingolipid system. Antidepressants accumulate as weak, lipophilic bases in acidic intracellular compartments and can displace ASM localized there from the inner lysosomal membrane. ASM then undergoes intralysosomal proteolytic digestion; thus, many antidepressants act as FIASMAs. ASM cleaves sphingomyelin to form ceramide. Therefore, the inhibition of this enzyme leads to a reduction in ceramide that has a positive effect on neurogenesis and behavior. The “backward effect” of ASM inhibition results in the slow accumulation of sphingomyelin in the lysosome and subsequently an increase in the abundance of ceramide in the ER. This effect induces autophagy, which reduces depression. Investigations of this signaling pathway have provided hints for a new group of rapidly acting antidepressants through the inhibition of SMSs. Both types of effects of antidepressants on ASM—the forward effect on neurogenesis and the backward effect on autophagy—explain important clinical characteristics of depression. A second important molecular target for antidepressants has recently been described: interference with TRKB, the receptor for BDNF. This mechanism also explains important characteristics of the disease.

Of the aforementioned unmet needs in the pharmacological treatment of depression (see Section 3), recent findings on sphingolipid-based molecular antidepressant mechanisms explain therapeutic latency and give hope for the development of new, rapid-acting antidepressants that simultaneously favorably affect the multisystem and whole-body nature of the disease.

## Data Availability

Data sharing not applicable.
